# miR-146a Inhibits Cell Growth, Cell Migration and Induces Apoptosis in Non-Small Cell Lung Cancer Cells

**DOI:** 10.1371/journal.pone.0060317

**Published:** 2013-03-26

**Authors:** Gang Chen, Ijeoma Adaku Umelo, Shasha Lv, Erik Teugels, Karel Fostier, Peter Kronenberger, Alex Dewaele, Jan Sadones, Caroline Geers, Jacques De Grève

**Affiliations:** 1 Department of Pathology, First Affiliated Hospital, Guangxi Medical University, Nanning, Guangxi, People's Republic of China; 2 Laboratory of Medical and Molecular Oncology, Department of Medical Oncology, Oncology Center, Universitair Ziekenhuis Brussel, Vrije Universiteit Brussel, Brussels, Belgium; 3 Laboratory of Molecular and Cellular Therapy, Vrije Universiteit Brussel, Brussels, Belgium; 4 Laboratory for Biotechnology, Department of Gezondheidszorg, Erasmushogeschool Brussel, Brussels, Belgium; 5 Department of Pathology, Universitair Ziekenhuis Brussel, Vrije Universiteit Brussel, Brussels, Belgium; H. Lee Moffitt Cancer Center & Research Institute, United States of America

## Abstract

Aberrant expression of microRNA-146a (miR-146a) has been reported to be involved in the development and progression of various types of cancers. However, its role in non-small cell lung cancer (NSCLC) has not been elucidated. The aim of this study was to investigate the contribution of miR-146a to various aspects of the malignant phenotype of human NSCLCs. In functional experiments, miR-146a suppressed cell growth, induced cellular apoptosis and inhibited EGFR downstream signaling in five NSCLC cell lines (H358, H1650, H1975, HCC827 and H292). miR-146a also inhibited the migratory capacity of these NSCLC cells. On the other hand, miR-146a enhanced the inhibition of cell proliferation by drugs targeting EGFR, including both TKIs (gefitinib, erlotinib, and afatinib) and a monoclonal antibody (cetuximab). These effects were independent of the EGFR mutation status (wild type, sensitizing mutation or resistance mutation), but were less potent compared to the effects of siRNA targeting of EGFR. Our results suggest that these effects of miR-146a are due to its targeting of EGFR and NF-κB signaling. We also found, in clinical formalin fixed paraffin embedded (FFPE) lung cancer samples, that low expression of miR-146a was correlated with advanced clinical TNM stages and distant metastasis in NSCLC (*P*<0.05). The patients with high miR-146a expression in their tumors showed longer progression-free survival (25.6 weeks in miR-146a high patients vs. 4.8 weeks in miR-146a low patients, *P*<0.05). miR-146a is therefore a strong candidate prognostic biomarker in NSCLC. Thus inducing miR-146a might be a therapeutic strategy for NSCLC.

## Introduction

Non-small cell lung cancer (NSCLC) comprises 75–85% of newly diagnosed lung cancers. Over 70% of NSCLC patients present with advanced disease, and the overall 5-year survival rate for NSCLC is only 16%. For early-stage or locally advanced lung cancer, surgery is the most effective treatment, and combination chemotherapy is the standard adjuvant approach. For stage III/IV NSCLC, platinum-based combined chemotherapy is the current standard of care, but with much room for improvement [1], [2]. Lung carcinogenesis is a multistep process, which results from activation of oncogenes and inactivation of tumor suppressor genes. The molecular mechanisms underlying the development of NSCLC are currently still poorly understood. Therefore, a better understanding of these mechanisms will be helpful to develop novel therapeutic targets and strategies for the treatment of human NSCLC [3], [4], [5].

In recent years, microRNAs (miRNAs) have received increasing attention in cancer research. These small, non-coding RNAs can inhibit target gene expression by binding to the 3′ untranslated region of target mRNA, resulting in either mRNA degradation or inhibition of translation. MiRNAs play important roles in many normal biological processes involving cell proliferation, differentiation, apoptosis, and stress resistance [6], [7]. However, studies have also shown that aberrant miRNA expression or mutation is correlated with the development and progression of cancers. The miRNAs can have oncogenic or tumor suppressor activities, and thus miRNAs are emerging as targets for cancer therapy [8]. In addition, miRNAs could be used as prognostic and diagnostic biomarkers in cancer.

The expression of miR-146a has been found to be up-regulated in papillary thyroid carcinoma [9], anaplastic thyroid cancer [10] and cervical cancer [11], which suggests miR-146a could work as an “oncogenic” miRNA in these cancers. Nevertheless, lower expression of miR-146a was reported in prostate cancer [12], pancreatic cancer [13] and gastric cancer [14], [15]. Therefore, the role of miR-146a can vary in different types of cancers. Additionally, miR-146a level has been found to correlate with metastatic progression in oral tumors [16]. Functionally, miR-146a-expressing cells show markedly impaired invasion and migration capacity relative to control cells in both breast cancer [17] and pancreatic cancer [13]. Taken together, these findings suggest that modulating miR-146a levels has therapeutic potential to suppress invasions and metastases. However, to our knowledge, no studies have evaluated the association between miR-146a and NSCLC cells. Furthermore, there is no data available on the effects of miR-146a on the biology of NSCLC cells. miR-146a can target EGFR, based on predicted base pairing by using miRBase analysis [18], which has been functionally confirmed in breast cancer [17], [19] and pancreatic cancer [13]. EGFR plays a critical role in NSCLC and the activity of EGFR tyrosine kinase inhibitors (TKIs) in NSCLC, especially in the presence of activating EGFR mutations [20]. The complex EGFR signal transduction pathway involves the Ras/MAPK cascade, phosphatidyl inositol 3-kinase (PI3K), signal transducer and activator of transcription (stat), and downstream protein kinase C (PKC) [21]. Additionally, EGFR activates NF-κB by phosphorylation of IκB [22]. Furthermore, miR-146a plays a role in regulating NF-κB [13], [17], [19], [23] in different malignancies, although NF-κB is not a direct target of miR-146a. In addition, the relationship between miR-146a and signaling of EGFR or NF-κB has not been elucidated.

The potential importance of this particular miRNA, miR-146a, came to our attention in experiments we performed and that antedated the discovery of its role in other malignancies or its relationship to the EGFR mRNA. In these unpublished experiments, we profiled miRNA expression in Ba/F3 cells transfected with wild type and mutant EGFR genes respectively, using a liquid bead-based array previously described [24]. Comparative miRNA profiles were obtained in base-line conditions and under treatment with EGFR TKIs. The platform was an in-house proprietary platform and included 425 miRNAs. miR-146a was the miRNA that most strongly correlated with the mutational status of EGFR, and had the largest variation in response to TKIs treatment in EGFR mutant NSCLC cells and BA/F3 cells transfected with mutant EGFR compared to EGFR wild type cells. At the same time the sequence homology with EGFR was identified [18].

Prompted by these results, we further investigated the effect of miR-146a on cell growth, apoptosis and motility of human NSCLC cells. In addition, the effects of a miR-146a mimic was examined when combined with EGFR TKIs (gefitinib, erlotinib, and afatinib) and a monoclonal antibody (cetuximab) specifically in NSCLC cell lines that are resistant to EGFR TKIs. Finally, the relationship between the miR-146a expression and clinical parameters and prognosis was also explored using formalin-fixed paraffin-embedded (FFPE) lung cancer samples.

## Materials and Methods

### Cell lines and reagents

The human NSCLC cell lines H358, H1650, H1975, HCC827 and H292 were obtained from the American Type Culture Collection (*ATCC, Netherlands*) and cultured as described previously [25], [26]. The EGFR-specific monoclonal antibody cetuximab (2 mg/ml), EGFR TKIs gefitinib and erlotinib, and a panHER inhibitor of EGFR, HER2 and HER4 kinases, afatinib (BIBW 2992, Boehringer Ingelheim GmbH), were prepared as described previously [25], [26].

### Re-expression and inhibition of miR-146a in NSCLC cells

NSCLC cells were seeded in a 24-well plate (2.5×10^4^ cells per well) or a 96-well plate (2.5×10^3^ cells per well) and incubated at 37°C for 24 hrs. The cells were then transfected with a miR-146a mimic, a miRNA mimic negative control, a miR-146a inhibitor, or a miRNA inhibitor negative control (*Ambion, Life Technologies Europe B.V.,Gent, Belgium*) respectively at a final concentration of 60 nmol/L using Lipofectamine^TM^ 2000 (*Cat. No. 11668-019,Invitrogen Merelbeke, Belgium*). The cells were transfected with the miRNA mimic or miRNA inhibitor daily. After 5 days of transfection, the cells were split and transfected daily again up to day 10 [13]. The EGFR specific siRNA was described previously [25], [27] (sequence: GCAAAGTGTGTAACGGAATAGGTAT). The EGFR specific siRNA was transfected into NSCLC cells with the same method as above.

### Tissue samples

We collected tissues from 101 consecutive patients (age range from 29 to 83 years; mean 68.3 years) who had undergone surgical resection or biopsy for NSCLC at the two institutions. A sample of non-affected normal lung tissue was also collected from 76 patients and used as paired control. All samples were processed for pathological examination. The study protocol was approved by the local Ethical Committees of First Affiliated Hospital, Guangxi Medical University, China and Universitair Ziekenhuis Brussel (UZ Brussel), Belgium. Written informed consents were obtained from all patients. The clinicopathological parameters are described in Table 1.

**Table 1:** Relationship of miR-146a level and clinicopathological variables in lung cancer tissues.

Variables	No. of patients	miR-146a (2^−ΔCq^±SD)	*t*	*P*
*Tissues*				
Cancer	101	5.20±0.67	3.555	<0.001
Adjacent normal lung	76	14.01±2.72		
*Tissues (paired)*				
Cancer	76	5.91±0.75	2.873	0.005
Adjacent normal lung	76	14.01±2.72		
*Gender*				
Male	44	5.40±0.73	0.271	0.787
Female	57	5.05±1.04		
*Age (years)*				
<60	32	5.11±1.45	0.09	0.929
≧60	69	5.25±0.71		
*pTNM stage*				
I–II	28	10.19±1.81	3.694	0.001
III–IV	73	3.29±0.44		
*Distal metastasis*				
Absent	35	8.83±1.53	3.468	0.001
Present	66	3.28±0.48		
*EGFR protein expression*				
1–199	13	4.89±2.69	0.815	0.422
200–400	15	2.63±1.12		
*EGFR gene amplification* a				
Negative	20	4.21±1.79	0.116	0.908
Positive	9	3.85±2.21		
*EGFR mutation*				
Wild type	23	3.68±1.58	0.587	0.562
Mutation b	6	5.72±3.11		

a FISH negative with no or low genomic gain (≤four copies of gene in >40% of the cells) and FISH positive with gene amplification, defined by the presence of tight gene clusters, a gene/chromosome per cell ratio ≥2, or ≥15 copies of the genes per cell in ≥10% of the analyzed cells or with high polysomy (≥four copies of the gene in ≥40% of the cells). Eight cases had high polysomy and one case had gene amplification. Information for the different variables was not available for all cases in this retrospective series.

b Five cases had E746-A750 deletion (exon 19) and one case had L858R point mutation (exon 21).

### RT-qPCR

For *in vitro* experiments, the RNA isolation, RNA normalization, and reverse transcription were as described previously [25], [27], [28]. For clinical FFPE tissue, blocks were sectioned at a thickness of 10 µm (3 sections for total RNA isolation). The tissue was dewaxed by xylene and ethanol. The total RNA was isolated from tumor sections using the miRNeasy FFPE Kit (*QIAGEN, KJ Venlo, Netherlands*) according to the manufacturer's instructions with modifications by changing the incubation time after mixing with proteinase K to 36 hrs at 55 °C, meanwhile, adding proteinase K every 12 hrs to maintain its concentration. Depending on the size of the tumor sample, the RNA concentration ranged from 20 ng/µl to 2 µg/µl detected by Nanodrop 2000 (*Wilmington, DE 19810 USA*). Intron-spanning RT-PCR primers specific for EGFR or GAPDH mRNA were described previously [25], [28], [29]. The primers for miR-146a and RNU6B were included in TaqMan^®^ MicroRNA Assays (*4427975-000468, Applied Biosystems, Life Technologies Europe B.V.,Gent, Belgium*). The reverse primers were also used in the reverse transcription step with TaqMan^®^ MicroRNA Reverse Transcription Kit (*4366596, Applied Biosystems, Life Technologies Europe B.V.,Gent, Belgium*), in a total volume of 10 µl. Real-time qPCR for EGFR mRNA was performed in the Roche LightCycler^®^ 1.5 instrument with SYBR green detection and melting curve analysis, as described previously [28] and for miRNA, Applied Biosystems PCR7900 was used. The target mRNA or miRNA abundance in each sample was normalized to its reference GAPDH or RUN6B as reported [25], [27], [30].

### Cell growth

Cell growth was assessed using a colorimetric tetrazolium (MTS) assay (*CellTiter96 AQueous One Solution Cell Proliferation Assay G3580, Promega, Madison, USA*). The miRNA mimic, the miRNA inhibitor or siRNA were transfected daily for 0, 5 and 10 days. For the experiments combining miR-146a mimic and other agents (EGFR TKIs and mAb), the miR-146a mimic was initially transfected into the cells, and subsequently the cells were cultured for 7 days. The other agents were added on the 7^th^ day, and cell culture was prolonged for another 3 days. The protocol was as described previously [25].

### Cell viability

To further confirm the data from the MTS assay, cell viability was detected by fluorimetric detection of resorufin (*CellTiter-Blue Cell Viability Assay, G8080, Promega, Madison, USA*) as described previously [25].

### Caspase-3/7 activity detection

Caspase-3/7 activity was measured using a synthetic rhodamine labeled caspase-3/7 substrate performed immediately after the detection of cell viability on the same wells as described previously [25].

### Fluorescent microscopy evaluation of cell apoptosis and morphology

The effects of miR-146a mimic, or inhibitor or EGFR siRNA on apoptosis and nuclear morphology in the cells were assessed by Hoechst 33342 and propidium iodide (PI) double fluorescent chromatin staining as described previously [25].

### Wound-healing assay

Cells were seeded in individual wells of a 6-well culture plate. Transfection was performed as above. Before transfection, a sterile 10 µl pipette tip was used to longitudinally scratch a constant-diameter stripe in the confluent monolayer. The medium and cell debris were aspirated away and replaced with 2 ml of fresh medium. Photographs were taken at 0, 36, 72 and 108 hrs after wounding. For statistical analysis, ten randomly selected fields along each wound were marked, and the area of the wound was measured and the average was calculated as the wound area of this wound. The wound-healing area = wound area of 0 hrs-wound area of 36, 72 or 108 hrs, respectively, wound-healing area was first compared to the wound area of 0 hrs, and finally compared to the mock control.

### Western blot analysis

After being treated for the indicated periods, the cells were washed with PBS and lysed in a buffer containing Tris/HCl (ph 7.6) 20 mM, NaCl 150 mM (ph 6.85), EDTA 1 mM (ph 8), TRITON-X 1%, Na-pyrophosphate 2.5 mM, Sodium orthovanadate (Na3VO4) 1 mM, Leupeptin 1 µg/ml, protease inhibitor cocktails 1% and phosphatase inhibitor cocktails 1% (*Sigma-Aldrich NV/SA, Bornem, Belgium*). The lysates were centrifuged at 12,000×g for 10 min at 4 °C and boiled for 5 min. The protein concentration of the lysate was detected by the Bio-Rad Bradford protein assay (*Nazareth Eke, Belgium*) and 25 µg of denatured protein was subjected to SDS-PAGE (10% SDS-acrylamide gel) with a loading buffer containing 80 mM Tris-HCl (ph 6.8), 5% SDS,10% glycerol, 5 mM EDTA (ph 8), 5% 2-MercaptoEthanol, 0.2% Bromophenolblue and 1 mM phenylmethylsulfonyl fluoride as described previously [25]. The membrane was incubated with the following primary antibodies as indicated: EGFR (*Cell Signaling*), phospho-EGFR (Tyr1173, clone 9H2, *Upstate*), phospho-ERK1/2 (pTpY185/187, *Invitrogen*), phospho-AKT/PKB (Ser473, *Invitrogen*), phospho-stat3 (Tyr705, 3E2, *Cell Signaling*), phospho-stat5 (Tyr694, *BD Biosciences*), phospho-IκBα (Ser32/36, 5A5, *Cell Signaling*), IκBα (L35A5, *Cell Signaling*), phospho-NF-κB p65 (Ser536, *Cell Signaling*), NF-κB (*Cell Signaling*), phospho-IRAK-1(Ser376, *Santa Cruz Biotechnology*), IRAK-1 (*Santa Cruz Biotechnology*) and β-actin (*Sigma-Aldrich N.V*.).

### Statistical analysis

SPSS19.0 was used for statistical analysis. Results were representative of three independent experiments unless stated otherwise. Values were presented as the mean±standard deviation (SD). One-way Analysis of Variance (ANOVA) test was used to analyze significance between groups. The Least Significant Difference (LSD) method of multiple comparisons with parental and control group was applied when the probability for ANOVA was statistically significant. Survival analysis was performed using the log-rank test and Kaplan-Meier plots approach. Statistical significance was determined at a *P*<0.05 level. In the analysis of additivity and synergism, the theoretical zero-interaction (exactly additive) dose-response curve for each miR-146a mimic+drug combination was calculated by applying Bliss independence criterion [31], [32]. The statistical evaluation of the additive or synergistic effect was done by comparing each miR-146a mimic+drug dose-response curve with the Bliss independence curve. Synergism is concluded when miR-146a mimic+drug dose-response curve is higher than the 95% confidence intervals to the respective Bliss independence curve, while additivity is when the miR-146a mimic+drug dose-response curve is lower than that of Bliss independence curve and higher than the individual treatment curve. The analysis of additivity and synergism was also assessed by the Biosoft CalcuSyn program (Ferguson, MO, USA). The Combination Index (CI) was used to express synergism (CI<1), additive effect (CI = 1), or antagonism (CI>1) [33].

## Results

### miR-146a inhibits cell growth and induces cell apoptosis in NSCLC cells

**Figure 1:**
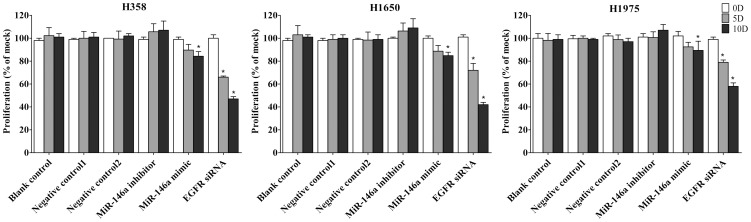
miR-146a inhibits cell growth of NSCLC cells by MTS assay. NSCLC cells (H358, H1650 and H1975) were incubated in the presence of miR-146a inhibitor, mimic, EGFR siRNA and different controls, for 0, 5 and 10 days. Cell growth was measured using the colorimetric tetrazolium (MTS) assay (CellTiter96 AQueous One Solution Cell Proliferation Assay). Negative control 1 is miRNA inhibitor negative control and Negative control 2 is miRNA mimic negative control. * *P*<0.05, compared to blank control at the same time point.

**Figure 2:**
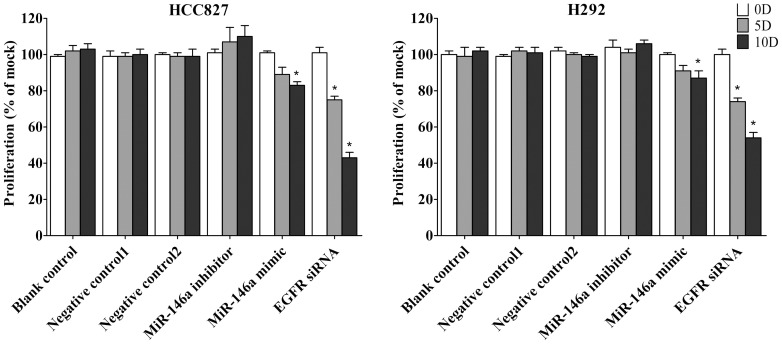
miR-146a inhibits cell proliferation with MTS in HCC827 and H292 cell lines. NSCLC cells were treated as mentioned in Figure 1. * *P*<0.05 compared to blank control at the same time point.

**Figure 3:**
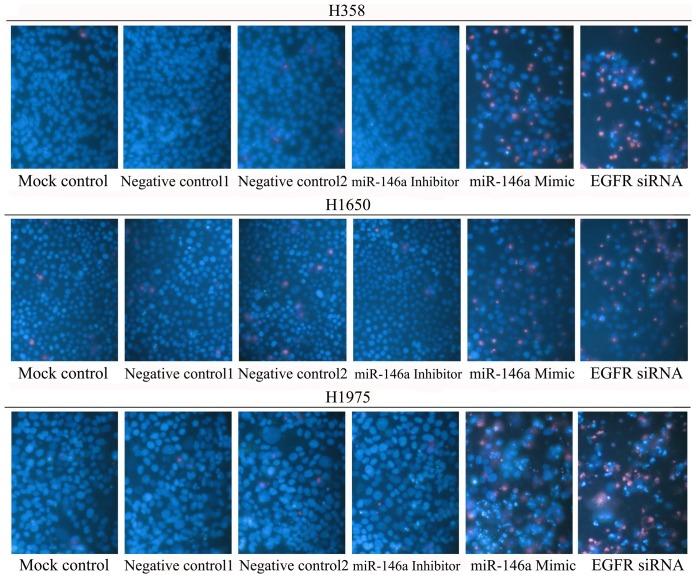
miR-146a suppresses cell growth and induces apoptosis with Hoechst 33342 and PI double fluorescent staining. NSCLC cells were treated as mentioned in Figure 1, and the effect on apoptosis was assessed and compared to the mock control at 0, 5 and 10 days post transfection with Hoechst 33342 and PI double fluorescent staining.

**Figure 4:**
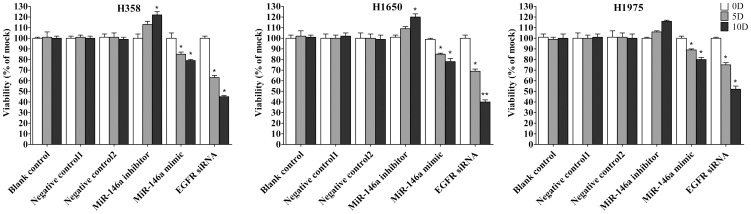
miR-146a suppresses cell growth with hoechst 33342 and PI double fluorescent staining. The effect of miR-146a on cell growth (H358, H1650 and H1975) was assayed with hoechst 33342 and PI double fluorescent staining. * *P*<0.05, ** *P*<0.01 compared to blank control at the same time point.

**Figure 5:**
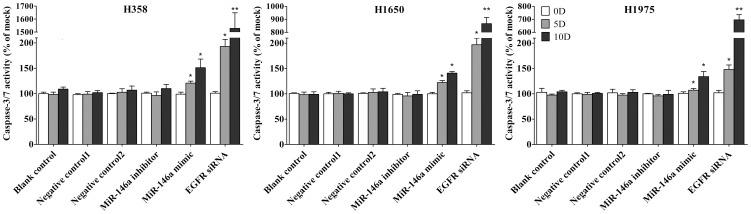
miR-146a increases cellular caspase-3/7 activity. NSCLC cells were treated as mentioned in Figure 1, and caspase-3/7 activity was examined and compared to mock controls. * *P*<0.05, ** *P*<0.01 compared to blank control at the same time point.

**Figure 6:**
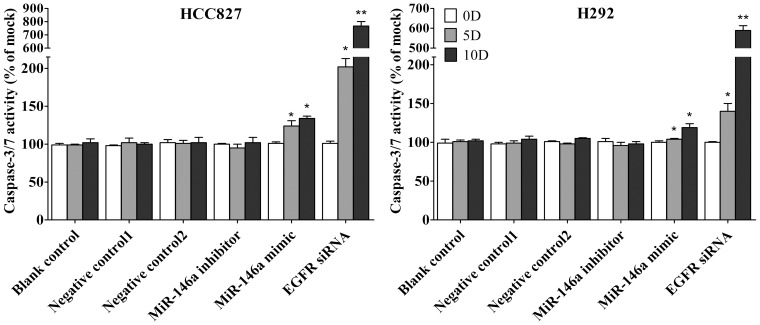
miR-146a increases caspase-3/7 activity in HCC827 and H292 cell lines. NSCLC cells (HCC827 and H292) were treated as mentioned above. * *P*<0.05, ** *P*<0.01 compared to blank control at the same time point.

**Figure 7:**
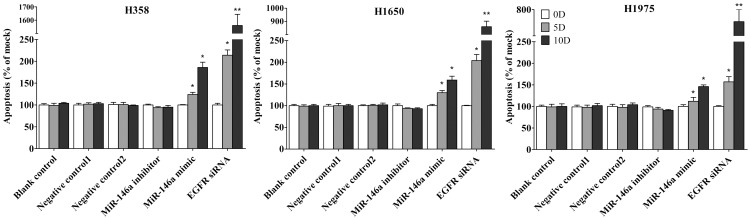
miR-146a induces apoptosis with hoechst 33342 and PI double fluorescent staining. The effect of miR-146a on apoptosis in H358, H1650 and H1975 was examined with hoechst 33342 and PI double fluorescent staining. * *P*<0.05, ** *P*<0.01 compared to blank control at the same time point.

First the base line expression of miR-146a was assessed in all the cell lines studied by real time RT-qPCR assay. Transfection efficiency of the miR-146a mimic and inhibitor was also first verified by RT-qPCR assay. The miR-146a expression level at 5 and 10 days post-transfection was analyzed. After transfection with the miR-146a inhibitor, ΔΔCq was 1.82 (71.68% miR-146a knock-down) for H358, 1.09 (53.02% knock-down) for H1650, 2.4 (81.05% knock-down) for H1975, 1.92 (73.57% miR-146a knock-down) for HCC827, and 1.89 (73.02% knock-down) for H292 10 days post-transfection. After transfecting the miR-146a mimic for 10 days, miR-146a levels were severely increased, with ΔΔCq −14.69 (26431.0372 folds) for H358, −10.97 (2005.8528 folds) for H1650, −12.78 (7032.3681 folds) for H1975, −13.25 (9741.9847 folds) for HCC827 and −13.81 (14362.3081 folds) for H292. Negative controls had no change of the level of miR-146a (data not shown). With the miR-146a inhibitor, cell proliferation was slightly enhanced in all cell lines tested, but without significant difference compared to mock controls. After transfection with the miR-146a mimic, a significant reduction in proliferation was noted on the 10^th^ day in all five cell lines, although less than what is observed with siRNA targeting EGFR (Figure 1, Figure 2). To verify these results, the effect on viability was assessed using a fluorimetric resorufin viability assay (CellTiter Blue, Promega, data not shown), and by microscopic counting of viable (Hoechst 33342 positive/PI negative) cells (Figure 3, Figure 4). In both assays the results largely mirrored the MTS tetrazolium assay results. To verify whether miR-146a is able to induce apoptosis, the CellTiter Blue assay was multiplexed with a fluorescent caspase 3/7 assay (Apo One, Promega). The results show that the miR-146a inhibitor did not increase caspase-3/7 activity. However, the miR-146a mimic significantly enhanced caspase-3/7 activity in all five NSCLC cell lines tested, but the effect was much less than what is seen with siRNA targeting EGFR (Figure 5, Figure 6). The effect on apoptosis was confirmed microscopically by Hoechst 33342 and PI double fluorescent staining (Figure 3, Figure 7).

### miR-146a suppresses the motility of NSCLC cells

**Figure 8:**
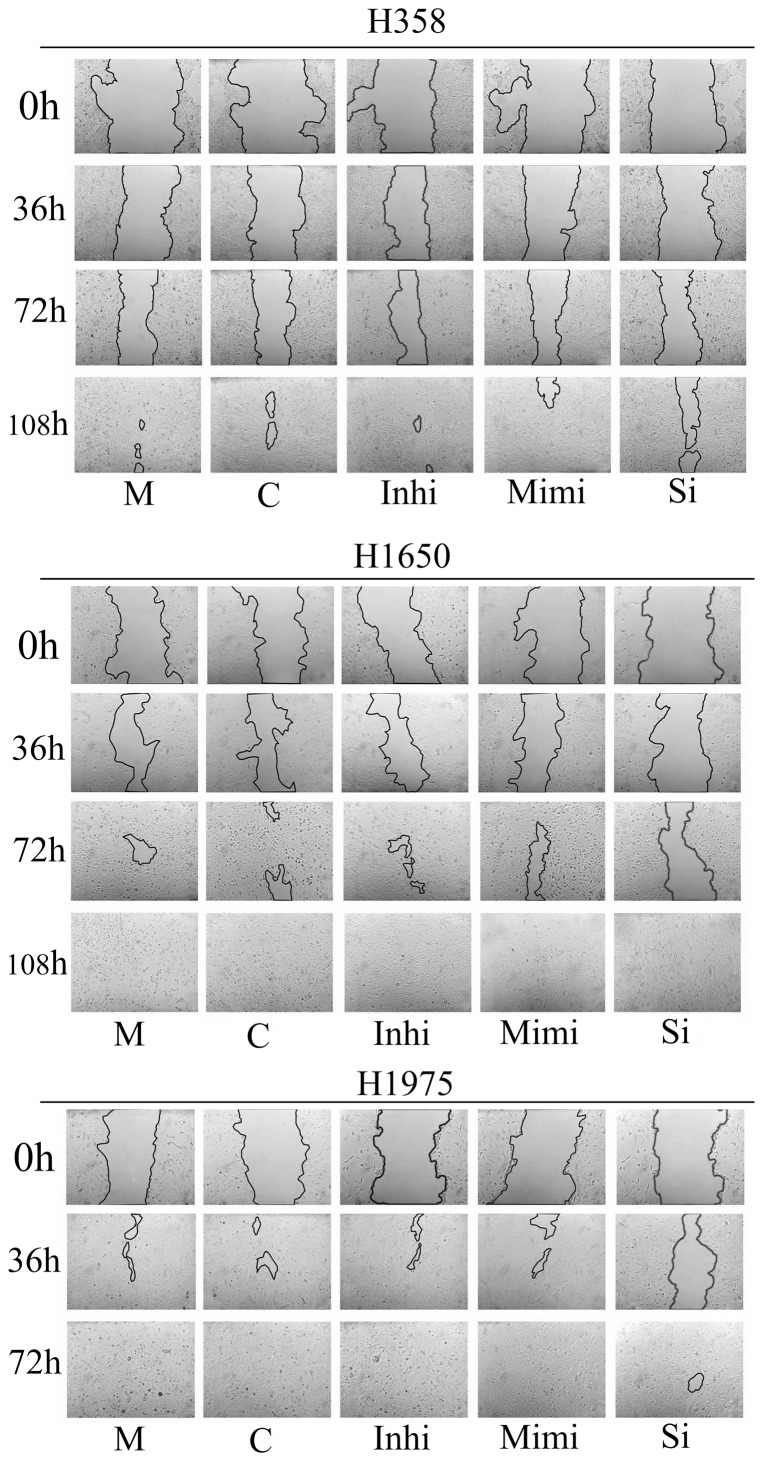
miR-146a inhibits motility in NSCLC cells. NSCLC cells were incubated in the presence of miR-146a inhibitor, miR-146a mimic, EGFR siRNA and different controls for 0, 36, 72 and 108 hrs post-transfection. Motility was detected using the wound-healing assay. The pictures from one field of a well representative of ten fields of vision in one well are shown (×100). M: mock control; C: Negative control for miRNA mimic; Inhi: miR-146a inhibitor; Mimi: miR-146a mimic; Si: EGFR-specific siRNA. The wound-healing rate is expressed relative to the mock control.

**Figure 9:**
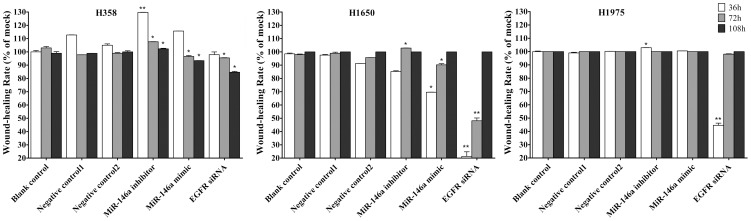
miR-146a inhibits motility in NSCLC cells. NSCLC cells were treated as in the experiments represented in Figure 8. The wound-healing rate is expressed relative to the mock control. The average ratio was calculated from ten fields and experiments were repeated in three independent wells. * *P*<0.05, **P<0.01 compared to blank control at the same time point.

Next, we evaluated the effect of miR-146a function on the motility of three NSCLC cell lines H358, H1650 and H1975. In the mock controls, untransfected H358 cells need the longest time to heal (>108 hrs). On the contrary, H1975 cells healed the fastest (<72 hrs). The miR-146a inhibitor caused a marked accelerated wound-healing rate in H358 cells, and had little effect in H1650 and H1975 cells. The miR-146a mimic led to a moderately decrease wound-healing rate in H358 and H1650 cells respectively. However, the miR-146a mimic had no influence on cell motility in H1975 cells. The siRNA targeting EGFR inhibited the wound-healing rate in all the cell lines tested, with a much stronger effect than the miR-146a mimic (Figure 8, Figure 9).

### miR-146a causes inhibition of EGFR and NF-κB signaling in NSCLC cells

**Figure 10:**
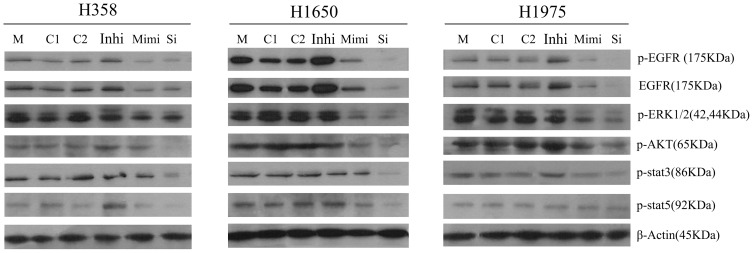
miR-146a reduces EGFR and its downstream signaling effectors in NSCLC cells. NSCLC cell lines were transfected with miR-146a inhibitor, miR-146a mimic, EGFR-specific siRNA and different negative controls and western blot was assayed 10 days post transfection. Antibodies included phospho-EGFR (p-EGFR), EGFR, p-ERK1/2, p-AKT, p-stat3, p-stat5 and β-Actin. M: mock control; C1: Negative control for miRNA inhibitor; C2: Negative control for miRNA mimic; Inhi: miR-146a inhibitor; Mimi: miR-146a mimic; Si: EGFR-specific siRNA.

**Figure 11:**
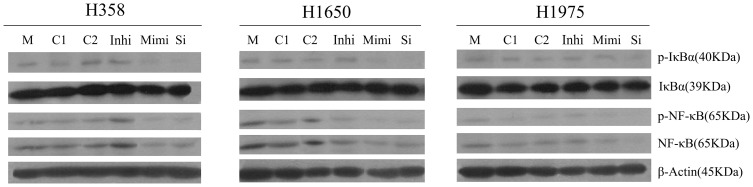
miR-146a reduces phosphorylation of NF-κB inhibitor IκBα in NSCLC cells. NSCLC cell lines were treated as in Figure 4. Antibodies included phospho-IκBα (p-IκBα), IκBα, p-NF-κB, NF-κB and β-Actin. M: mock control; C1: Negative control for miRNA inhibitor; C2: Negative control for miRNA mimic; Inhi: miR-146a inhibitor; Mimi: miR-146a mimic; Si: EGFR-specific siRNA.

**Figure 12:**
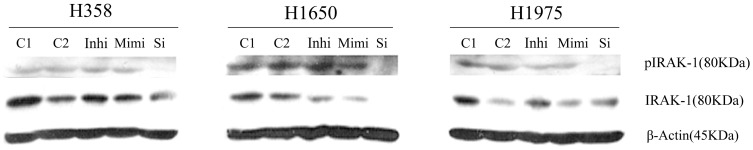
miR-146a downregulates IRAK-1 in NSCLC cells. NSCLC cell lines were treated as in Figure 4. Antibodies included phospho-IRAK-1 (S^376^), IRAK-1and β-Actin. C1: Negative control for miRNA inhibitor; C2: Negative control for miRNA mimic; Inhi: miR-146a inhibitor; Mimi: miR-146a mimic; Si: EGFR-specific siRNA.

To investigate the role of miR-146a in the regulation of cellular signaling, we transfected NSCLC cells with miR-146a inhibitor and mimic, and cultured the cells for a period of up to 10 days. We found that the increased expression of miR-146a in the NSCLC cells resulted in the down-regulation of EGFR both at the mRNA (data not shown) and protein levels (Figure 10). Importantly, we found that phosphorylated EGFR was also downregulated after miR-146a mimic transfection similar to EGFR-specific siRNA in different cell lines (Figure 10). At the same time, downstream signaling (AKT, ERK and stat pathways) was also down-regulated by miR-146a mimic, with a weaker effect as compared to EGFR-specific siRNA. To confirm the effects of miR-146a on EGFR, we transfected the NSCLC cells with the miR-146a inhibitor and found that inhibition of miR-146a increased the levels of p-EGFR, EGFR and downstream signaling (Figure 10). These results support the inhibitory effects of miR-146a on EGFR autophosphorylation and downstream signaling. Because miR-146a was reported to also regulate the expression of IRAK-1, which can activate NF-κB signaling [13], [34], [35], we investigated the effects of miR-146a on NF-κB signaling pathways in the NSCLC cell lines. miR-146a mimic reduced phosphorylation of the NF-κB inhibitor IκBα, but not the total IκBα. Moreover, the level of phospho-NF-κB, total NF-κB and total IRAK-1 were also decreased after miR-146a mimic transfection (Figure 11, Figure 12), indicating that miR-146a regulates NF-κB and IRAK-1 signaling.

### miR-146a mimic enhances the cell proliferation inhibitory effect of TKIs and cetuximab

**Figure 13:**
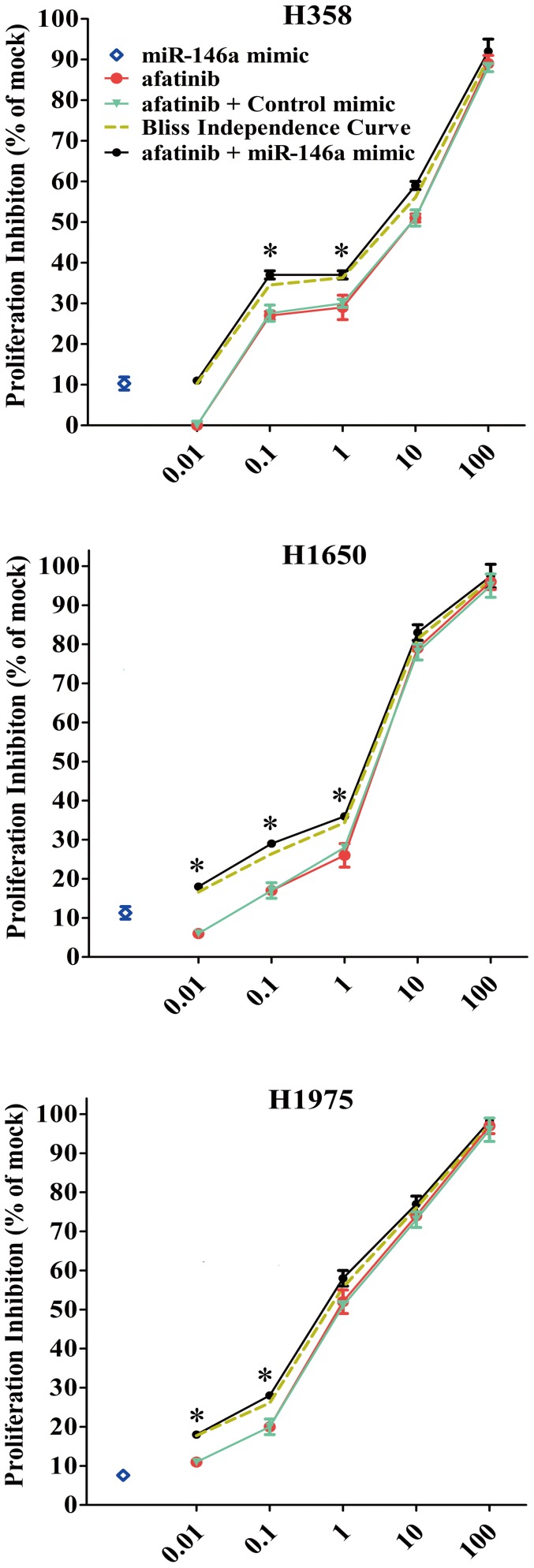
miR-146a enhances the growth inhibitory effect of afatinib in NSCLC cell lines. MTS was performed as above and the proliferation inhibition rate was calculated. * *P*<0.05, compared to agent alone. Bliss independence criterion was performed to calculate the theoretical additive effect. - - -- - -, bliss independence curve, which indicates the theoretical situation in which the combined effect of miR-146a mimic and other agent is exactly additive. •, drugs+miR-146a mimic. ▾drugs+Negative control mimic. ▪ drugs alone. ◊miR-146a mimic alone (60 nM). Data points represent mean values from triplicate wells, and error bars are S.D.

In earlier work we have examined the effects of combing EGFR siRNA with EGFR TKIs or cetuximab. Among all the agents tested, afatinib, an ErbB family inhibitor, had the strongest growth inhibitory effect [25]. We sought to investigate how this would compare to the effect of combining the miR-146a mimic and TKIs (gefitinib, erlotinib, afatinib) or cetuximab, using the colorimetric MTS formazan proliferation assay. The inhibition of cell proliferation was much higher when miR-146a mimic was combined with afatinib, compared to single drug or single miR-146a mimic in all tested cell lines, especially at lower, sub-molar concentrations of afatinib. However, not the whole cell growth curve of the proliferation was higher than the Bliss independence curve, which indicated the combination effect was additive (Figure 13). The combination effects of miR-146a mimic with other TKIs or cetuximab were similar but weaker than that of afatinib (data not shown). Thus miR-146a mimic enhances the cell proliferation inhibitory effect by TKIs and cetuximab. To verify the additive or synergistic nature of combining TKI/cetuximab with the miR-146a mimic, a CI was calculated [31], [32]. This unambiguously shows that the effect is additive (data not shown).

### Initial exploration of the clinical significance of miR-146a expression in NSCLC cases

**Figure 14:**
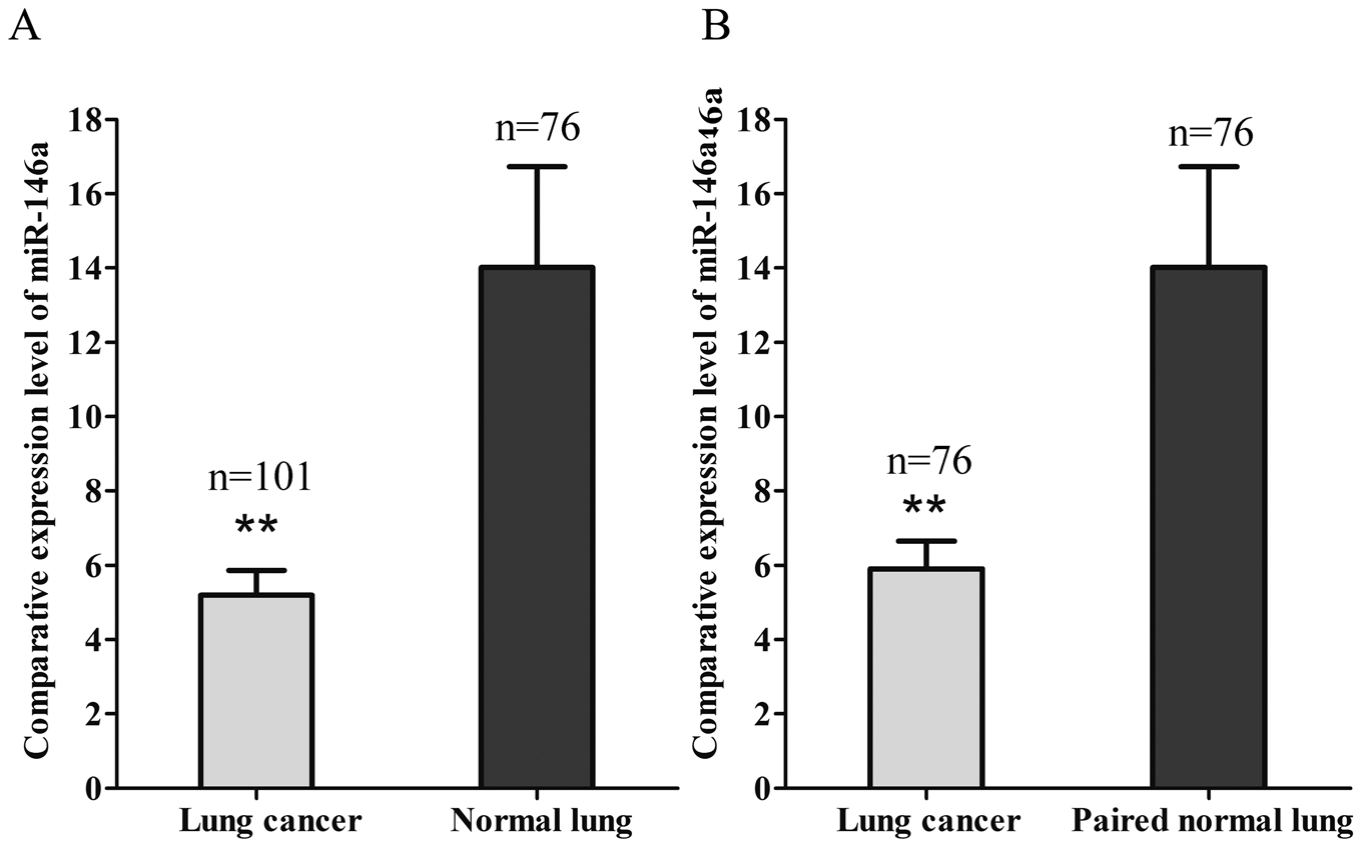
miR-146a expression between NSCLC samples and normal lung tissues. miR-146a levels (normalized to RNU6B) accessed by RT-qPCR in 101 cases of NSCLC cancer tissues and 76 cases of non-cancerous lung tissues (Panel A). miR-146a expression was compared in the paired 7 cases. * *P*<0.05.

**Figure 15:**
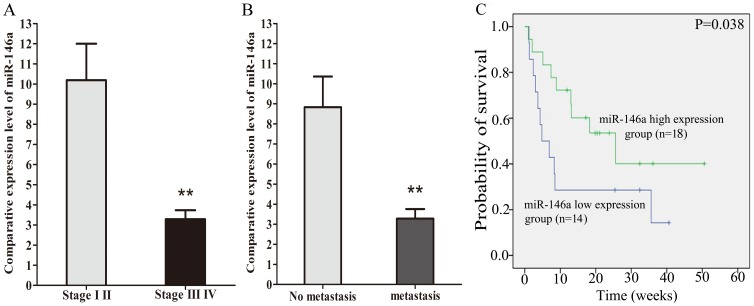
miR-146a expression and prognosis in thirty-two NSCLC cases. miR-146a levels accessed by RT-qPCR in stages I and II vs stages III and IV (Panel A), with no distant metastasis and with metastasis (Panel B). Kaplan-Meier overall survival curves according to miR-146a level in Panel C.

We next examined the miR-146a expression in FFPE biopsies from 101 cases of NSCLC. The biopsies were obtained before any systemic treatment. In the series, 76 cases were available with corresponding adjacent normal lung tissues. The relative miR-146a expression was overall significantly lower in NSCLC tissues than in the normal lung tissues (5.20 vs 14.01, *P*<0.001) (Figure 14). In the series of 76 patients with a paired sample, there was also a significantly lower miR-146a expression in cancer tissues compared to normal lung (5.91 vs 14.01, *P* = 0.005, Figure 14). miR-146a levels were significantly higher (*P* = 0.001) in the cases with a clinical TNM stages I and II compared to stages III and IV (Table 1, Figure 15A). Moreover, the miR-146a expression was significantly higher (*P* = 0.001) in the cases without distant metastasis than those with metastasis (Table 1, Figure 15B). Furthermore, the 32 patients with higher miR-146a expression (higher than the average level) had a longer progression-free survival than the patients with a low expression. Overall survival time of patients with high miR-146a expression was longer than that of patients with low expression (25.6 weeks in miR-146a high patients vs. 4.8 weeks in miR-146a low patients, *P* = 0.038, Figure 15C). The miR-146a expression was not related to other clinicopathological parameters, such as gender, age, EGFR protein expression, EGFR gene amplification or EGFR mutation status (Table 1).

## Discussion

In the current work we have explored the role of miR-146a in human NSCLCs. First we independently identified miRNA-146a as the strongest correlated miRNA with EGFR activation status and pharmacological EGFR inhibition in a screen that involved an array of 425 miRNAs in a series of NSCLC cell lines and BA/F3 transfectants with various EGFR genomic status (unpublished data on file). This prompted the current investigation to confirm these findings and further explore the role of this miRNA in NSCLC. Functionally, miR-146a suppressed cell growth, inhibited cell migration, induced cell apoptosis and inhibited EGFR downstream signaling in NSCLC cell lines. Furthermore, miR-146a enhanced the inhibition of cell proliferation caused by drugs targeting EGFR, including TKIs (gefitinib, erlotinib, and afatinib) and a monoclonal antibody (cetuximab). We also found in clinical FFPE samples that low expression of miR-146a was correlated with advanced clinical TNM stages and distant metastasis in NSCLC. The patients with high miR-146a expression showed longer progression-free survival.

### EGFR is a target gene of miR-146a

Initially we studied the miRNA expression profile in a controlled model (Ba/F3 cells) in which we expressed the EGFR wild type and kinase domain mutant forms. In that model we examined the miRNA expression profile in conditions of EGFR inhibition with TKIs. These experiments were performed before the role of miR-146a in other cancer types was published. From that work it appeared that miR-146a was the miRNA that correlated the most with EGFR function and provided the strongest correlation with the effects of TKIs among all 425 miRNAs evaluated. These data were then confirmed by RT-qPCR (data on file, not shown). We subsequently also treated NSCLC cells with EGFR siRNA, TKIs or mAb. When EGFR is downregulated by siRNA, TKIs (gefitinib, erlotinib or afatinib) or cetuximab, the miR-146a expression was up-regulated in NSCLC cells (data on file, not shown). These data together with an examination of the miRBase [18] provided evidence that EGFR is a target gene of miR-146a and with potential functional significance in lung cancer and EGFR signaling. Therefore, we wanted to confirm and study further the functional role of miR-146a in lung cancer.

### miR-146a is a tumor suppressor miRNA in NSCLC

miR-146a has been implicated, both in the development of various cancers and in the negative regulation of inflammation induced through the innate immune response [9], [36]. The polarity of the implication of miR-146a in cancer differs however between a tumor suppressor versus an oncogenic role in various tumor types. Overexpression of miR-146a has been found in papillary thyroid carcinoma [9] and anaplastic thyroid cancer [10] compared to unaffected normal thyroid tissues. The up-regulation of miR-146a was also detected in cervical cancer tissues [11]. Under-expression of miR-146a has been found to characterize progression to metastatic oral tumors [16]. Conversely reduced miR-146a expression was associated with hormone-refractory prostate cancer [12]. Li et al [13] reported a lower expression of miR-146a in pancreatic cancer cells compared with normal human pancreatic duct epithelial cells. Kogo et al [14] and Hou et al [15] also reported that miR-146a has a potential as a novel suppressor microRNAs in gastric cancer. Boominathan et al regarded miR-146a as a tumor suppressor miRNA [37]. Therefore, the role of miR-146a seems to vary in different types of cancers. These contrasting roles may be explained by the differences regarding the targets repressed by miR-146a in each tissue. For instance, tumors of papillary thyroid carcinoma [9] in which the up-regulation of miR-146a was strongest showed dramatic loss of KIT transcript and Kit protein, which indicates that up-regulation of miR-146a and regulation of KIT are involved in papillary thyroid carcinoma pathogenesis. NF-κB is also reported to contribute to anaplastic thyroid cancer and breast cancer up-regulating the expression of miR-146a [10], [17]. miR-146a may modulate HA/ROCK1-mediated tumorigenecity in androgen-dependent prostate cancer [12]. In both pancreatic cancer and gastric cancer cells, EGFR and the NF-κB regulatory kinase interleukin 1 receptor-associated kinase 1 (IRAK-1) were proven to be.miR-146a target genes [13], [14]. Boominathan et al reported that miR-146a expression can be up-regulated by p53 and TA-p73/p63 [37]. Thus, miR-146a may be associated with a complex network of gene expression regulation that could be tissue and stage dependent, targeting different mRNA species in each circumstance. The association between miR-146a and NSCLC had not yet been examined before the initiation of the current study. In the present study, we explored the role of miR-146a in NSCLC, using five cell lines, one with a KRAS mutation and EGFR wild type (H358), one with KRAS and EGFR wild type (H292), and three cell lines with a EGFR kinase domain mutation. H1650 and HCC827 have an in-frame deletion in the EGFR tyrosine kinase domain (ΔE746-A750, exon 19). H1650 cells have also a deletion of the 3′ part of exon 8 and the entire exon 9 of PTEN, which causes loss of the protein [38]. The cell line H1975 has a sensitizing L858R kinase domain mutation in exon 21, but also a second mutation (T790M, *in cis*, in the kinase domain) rendering them resistant to the reversible TKIs gefitinib and erlotinib [39]. miR-146a was expressed in all the five cell lines studied, with the similar magnitude, independent of the genomic status of the EGFR. Concurrent to our results, most recently, Vince et al [40] found that the expression of miR-146a could be detected in 101 cases of NSCLC and their corresponding paired non-affected control lung tissues. We also found miR-146a expression in a series of one hundred and one FFPE lung cancer biopsy samples obtained prior to systemic therapy and seventy-six pairs of their corresponding adjacent normal lung tissues by real time RT-qPCR. miR-146a expression was significantly lower in lung cancer tissues than normal lung tissues with a similar trend in the series of matched tissues. These data support a suppressor function for miR-146a in lung cancer. This is in contrast to results of Vince et al [40], who found that the relative expression of miR-146a was not significantly different between NSCLC and their corresponding paired non-affected control lung tissues. miR-146a is located on chromosome 5q33.3 [41], [42], [43] and copy number gains on chromosomes 5q occurs more frequently in non-smokers compared to smokers in NSCLC patients [44]. The smoking history of the patients included in our study was not available for all samples and therefore we cannot at this stage comment about the potential differential role of miR-146a in lung cancer occurring in non-smokers versus smokers. A larger cohort of patients, preferably collected in a prospective trial with more detailed clinical annotation is needed to further confirm the role of miR-146a in NSCLC in a clinical context. To estimate whether this would be likely a tumor suppressor (as suggested by the retrospective clinical cohort studied) or as an oncogene, we performed a series of *in vitro* studies.

### Effect of miR-146a on cell growth and apoptosis in NSCLC

We examined the functional significance of miR-146a in NSCLC *in vitro*, both by inhibiting (with an inhibitor) or simulating enhanced expression of miR-146a with a mimic. We found that overexpression of miR-146a caused decreased cell growth, and increased cellular apoptosis. The down-regulation of the EGFR and its downstream pathway (ERK-1/2, AKT and stat) by miR-146a mimic explains the mechanism for miR-146a to inhibit cell growth and induce apoptosis in NSCLC. Our results collectively support a tumor suppressive role of miR-146a in NSCLC. However, the down-regulation of the EGFR and downstream pathway by miR-146a mimic was less potent compared to siRNA targeting EGFR and also lead to a weaker effect on cell growth and apoptosis compared to what we observed with EGFR-specific-siRNAs. The effect of miR-146a mimic was not significantly different in the five studied cell lines, which could suggest that the sensitivity to miR-146a mimic in NSCLC cells may have no important relationship with EGFR genomic status. It should be noted however that miR-146a mimic did have a weaker effect in H1975 cells with regard to cell growth inhibition and apoptosis induction. This cell line also has less sensitivity to EGFR siRNA treatment. This indicates that T790M mutant EGFR is a less potent driver of cell growth and survival compared to mutant EGFR without a T790M. This could be an additional explanation for the clinical resistance to TKI inhibition of that receptor, even when the covalent binding ErbB family inhibitor afatinib is used. Others also have found suppressive effects of miR-146a on the malignant phenotype of other cancer types. Functional studies showed that, when introduced into cell lines, miR-146a was found to promote cell proliferation in cervical cancer cells [11], which suggests that miR-146a works as an oncogenic miRNA in these cancers.

### Effect of miR-146a on cell motility in NSCLC

The relationship between miR-146a and tumor motility, invasion and metastases were reported by several groups. Bhaumik et al [17] demonstrated that miR-146a is expressed in the highly metastatic human breast cancer cell line MDA-MB-231. Functionally, lentiviral-infected miR-146a MDA-MB-231 cells showed markedly impaired invasion and migration capacity relative to control cells without being lentiviral-infected. Li et al [13] reported that re-expression of miR-146a inhibited the invasive capacity of pancreatic cancer cells with concomitant down-regulation of EGFR and IRAK-1. To investigate the relationship between miR-146a and the mobile capacity in NSCLC, we performed wound-healing assays. Our experiments indicate that miR-146a has a differential effect on the motility/invasion properties of the NSCLC lines studied. Among all the cell lines tested, H1975 showed the fastest wound-healing rate, identifying this cell line as highly invasive. We have also observed this using another assay [45]. A weak inhibitory effect of miR-146a on motility of H358 cells that are EGFR wild type was observed, while there was an important effect on the EGFR mutant H1650 cells. These results are consistent with recent reports in pancreatic and breast cancers [13], [17]. In contrast, the invasive properties of the H1975 cells seemed independent of miR-146a expression. The T790M mutation in this cell line might be a possible mechanism for resistance to the miR-146a mimic in motility, due to the sequence change, It is also possible that the EGFR T790M mutant is less important for the invasive phenotype, as we also surmised for the lesser effect on growth and apoptosis in that cell line. We also observed decreased levels of phosphorylated IκBα, phosphorylated NF-κB and total NF-κB after enhanced expression of miR-146a, suggesting that miR-146a can regulate IκB/NF-κB signaling that contributes to cell motility and migration in NSCLC cells. From our results, we believe that miR-146a inhibits cell motility and migration partly through regulation of EGFR, NF-κB and IRAK-1 signaling (Figure 10, Figure 11, Figure 12). The amplitude of the motility inhibition by miR-146a mimic is again much lower than by siRNA targeting wild type EGFR as it was with regard to proliferation and cell survival. Also here, the less inhibition of EGFR and NF-κB pathways, as showed by western blot could explain that result.

### Therapeutic potential of the miR-146a mimic in NSCLC

The suppressive role of miRNA on the malignant phenotype of lung cancer cells makes it tempting to speculate that novel strategies by which one could induce higher levels of miR-146a in cancer cells could be explored therapeutically. When we combined the miR-146a mimic together with different TKIs or cetuximab in NSCLC cells, miR-146a was found to enhance the cell proliferation inhibition by TKIs and cetuximab, the strongest effect obtained when combined with afatinib. The level of miR-146a in cancer cells can also be up-regulated by other agents. For example, Li et al found that re-expression of miR-146a by nontoxic ‘‘natural agents’’, including 3,3′-diinodolylmethane and isoflavone has anti-tumor effects in pancreatic cancer [13].

### Clinical significance of miR-146a expression in NSCLC cases

Our *in vitro* data support the potential clinical relevance of our observations in patient samples that indicate that miR-146a is downregulated in malignant versus normal lung tissue and that expression of miR-146a inversely correlates with stage and outcome of patients. This should be confirmed in a larger prospectively and clinically annotated cohort of NSCLC patients.

## Conclusions

Taken together, our preclinical and clinical results identify miR-146a as a novel tumor suppressor gene in NSCLC, involved in cell growth, cell survival and motility which can affect the aggressiveness of the disease and ultimately the outcome of the patient. miRNA-146 might thus be a potential prognostic marker for NSCLC, but needs to be confirmed in a larger clinical cohort. In addition, miR-146a also has a potential as a molecular therapeutic target. Further studies are needed to establish whether miR-146a or agents that can increase miR-146a level could be useful for the treatment of NSCLC.
